# 

**Published:** 1911-06

**Authors:** 


*The following donations have been received since the publication of the List in March.*

*May* 31*st*, 1911.

| | |
|---|---|
| American Urological Association (1) .. .. | 1 volume. |
| E. W. Hey Groves (2) .. .. .. .. | 2 volumes. |
| L. M. Griffiths .. .. .. .. .. | 4 ,, |
| R. Shingleton Smith, M.D. (3) .. .. .. | 1 volume. |

Mr. Stephen Paget (per Dr. Shingleton Smith) has given an unframed picture of his distinguished father, Sir James Paget, Bart.

### EIGHTIETH LIST OF BOOKS.

The titles of books mentioned in previous lists are not repeated.

The figures in brackets refer to the figures after the names of the donors, and show by whom the volumes are presented. The books to which no such figures are attached have either been bought from the Library Fund, or received through the *Journal*.

| | | |
|---|---|---|
| **Cattell, H. W.** .. .. | *Lippincott's New Medical Dictionary* .. .. | 1910 |
| **Corner, E. M.** .. .. | *The Life-History, Function, and Inflammation of the Appendix* .. .. .. .. .. | 1911 |
| **Darier, A.** .. .. | *Ophthalmic Therapeutics.* (Tran. by S. Stephenson) .. .. .. .. .. .. .. .. | 1911 |
| **Ehrlich, P.** .. .. | *Abhandlungen über Salvarsan* .. .. (2) | 1911 |
| **Ehrlich and S. Hata, P.** | *The Experimental Chemotherapy of Spirilloses.* (Tr. by A. Newbold and revised by R. W. Felkin) .. .. .. .. .. .. | 1911 |

**Hata, P. Ehrlich and S.** *The Experimental Chemotherapy of Spirilloses.* (Tr. by A. Newbold and revised by R. W. Felkin) .. .. .. .. .. .. 1911
**Hewlett, R. T.** .. .. *Serum and Vaccine Therapy* .. 2nd Ed. 1910
**Hutchinson, Sir F. Treves and J.** *Student's Handbook of Surgical Operations* .. .. .. .. .. 3rd Ed. 1911
**Jones, D. W. C.** .. *Introduction to Therapeutic Inoculation* .. 1911
**Kelynack, T. N.** [Ed.] *The Drink Problem* .. .. .. .. .. (3) [1907]
**Kerr, J. M. M.** .. .. *Operative Midwifery* .. .. .. .. .. .. 1908
**Low, R. C.** .. .. *Carbonic Acid Snow* .. .. .. .. .. .. 1911
**Macilwaine, S. W.** .. *Medical Revolution* .. .. .. .. .. .. 1911
**May, P.** .. .. .. *The Chemistry of Synthetic Drugs* .. .. 1911
**Miles, A. Thomson and A.** *Manual of Surgery* .. 2 vols. 3rd Ed. 1909
**Miller, R.** .. .. .. *The Medical Diseases of Children* .. .. 1911
*New and Unofficial Remedies* .. .. .. .. .. .. .. .. .. .. .. 1911
**Shenton, E. W. H.** .. *Disease in Bone and its Detection by the X-rays* .. .. .. .. .. .. .. .. 1911
**Smith, J. S. K.** .. *Lateral Curvature of the Spine and Flat-foot* 1911
**Sutton, J. B.** .. .. *Tumours, Innocent and Malignant* 5th Ed. 1911
**Thomson and A. Miles, A.** *Manual of Surgery* .. 2 vols. 3rd Ed. 1909
**Treves and J. Hutchinson, Sir F.** *Student's Handbook of Surgical Operations* .. .. .. .. .. 3rd Ed. 1911
**Vittoz, R.** .. .. .. *Treatment of Neurasthenia.* (Trans. by H. B. Brooke) .. .. .. .. .. .. .. 1911
**Walker, N.** .. .. *An Introduction to Dermatology* .. 5th Ed. 1911
**Webster, J. C.** .. .. *Female Pelvic Anatomy* .. .. .. .. .. 1892
**Wechselmann, W.** .. *The Treatment of Syphilis with Salvarsan.* (Trans. by A. L. Wolbarst) .. .. .. [1911]

### TRANSACTIONS, REPORTS, JOURNALS, &c.

American Association of Genito-Urinary Surgeons, Transactions of the .. .. .. .. .. .. .. .. .. .. .. .. .. .. Vol. V 1910
American Journal of Obstetrics, The .. .. .. .. .. Vol. LXII 1910
American Journal of Ophthalmology, The .. .. Vol. XXVII 1910
American Journal of Surgery .. .. .. .. .. .. Vol. XXIV 1910
American Medicine .. .. .. .. .. .. .. .. .. .. Vol. XVI 1910
American Pediatric Society, Transactions of the .. Vol. XXII 1910
American Surgical Association, Transactions of the Vol. XXVIII 1910
American Urological Association, Transactions of the (1) Vol. IV 1910
Annales de Dermatologie .. .. .. .. .. 5th ser., Tom. I 1910
Archiv für klinische Chirurgie .. .. .. .. Bds. XCII, XCIII 1910
Archives of Pediatrics .. .. .. .. .. .. .. .. Vol. XXVII 1910
Association of American Physicians, Transactions of the Vol. XXV 1910
Boston Medical and Surgical Journal, The .. .. .. .. Vol. CLXIII 1910
British Journal of Children's Diseases, The .. .. .. Vol. VII 1911
British Journal of Dermatology, The .. .. .. .. .. Vol. XXII 1910
British Journal of Tuberculosis .. .. .. .. .. .. Vol. IV 1910
Bulletin de l'Académie de Médecine .. .. Tom. LXIII, LXIV 1910

| | | |
|---|---|---|
| Bulletin de l'Academie royale de Médecine | Tom. XXIV | 1910 |
| Bulletin de la Société française de Dermatologie | | 1910 |
| Deutsche Zeitschrift für Chirurgie | Bds. CV–CVII | 1910 |
| Écho medical du Nord, L' | | 1910 |
| Gazette hebdomadaire des Sciences medicales de Bordeaux | Tom. XXXI | 1910 |
| Hospitals and Charities, Burdett's | | 1910 |
| Hospitals— | | |
| Johns Hopkins Hospital Reports | Vol. XVI | 1911 |
| St. Bartholomew's Hospital Reports | Vol. XLVI | 1911 |
| Indian Medical Gazette, The | Vol. XLV | 1910 |
| International Clinics | 2nd ser., Vols. I–III | 1893 |
| Jahreskurse für arztliche Fortbildung | (2) | 1910 |
| Johns Hopkins Hospital Bulletin, The | Vol. XXI | 1910 |
| Journal of Experimental Medicine, The | Vol. XII | 1910 |
| Journal of Hygiene | Vol. X | 1910 |
| Journal of Laryngology, The | Vol. XXV | 1910 |
| Journal of Medical Research, The | Vol. XXIII | 1910 |
| Journal of Mental Science, The | Vol. LVI | 1910 |
| Journal of Nervous and Mental Disease, The | Vol. XXXVII | 1910 |
| Journal of Obstetrics and Gynæcology, The | Vol. XVIII | 1910 |
| Journal of the Royal Sanitary Institute | Vol. XXXI | 1910 |
| Journal of Tropical Medicine, The | Vol. XIII | 1910 |
| Laboratory Reports of the Royal College of Physicians of Edinburgh | Vols. X, XI | 1911 |
| Library Association Record, The | Vol. XII | 1910 |
| Library, The | 3rd ser., Vol. I | 1910 |
| Medical Chronicle, The | 4th ser., Vol. XX | 1911 |
| Medical Library and Historical Journal | Vol. V | 1907 |
| Medical Libraries | Vols. III–V | 1901–2 |
| Montreal Medical Journal, The | Vol. XXXIX | [1910] |
| Parasitology | Vol. III | 1910 |
| Progrès médical, Le | | 1910 |
| Progressive Medicine | Vol. I | 1911 |
| Revue de Chirurgie | Bd. XLII | 1910 |
| Revue de Gynécologie | Tom. XIV | 1910 |
| Revue de Thérapeutique | | 1910 |
| Revue générale d'Ophtalmologie | Tom. XXIX | 1910 |
| St. Peterburger medicinische Wochenschrift | | 1910 |
| Smithsonian Institution, Report for | | 1909 |
| Surgery, Gynecology and Obstetrics | Vol. XI | 1910 |
| Therapeutic Gazette, The | Vol. XXXIV | 1910 |
| Univ. of Penna. Medical Bulletin | Vol. XXIII | 1911 |
| Zeitschrift für Urologie | Bd. IV | 1910 |
| Zentralblatt für Chirurgie | | 1910 |
| Zentralblatt für Gynäkologie | | 1910 |

the saddle without help, and also evident in his unique fielding close in at "point."

He was a remarkable man, and it will be many years before his words and deeds are forgotten.

He was married four times—in 1868, 1885, 1902 and 1907. There were thirteen children by the first wife, of whom six survive, and five by the second, of whom three survive.

### Local Medical Notes.

**University of Bristol.**

EXAMINATION RESULTS.

M.B., B.C. CANTAB.—*Surgery:* P. A. Opie.

M.B., B.S. LOND.—*First Examination for Medical Degrees:* W. K. A. Richards. *Second Examination for Medical Degrees—Part I:* G. M. Jackson, Mary E. Joll, D. G. C. Tasker; *Part II:* C. C. Harrison, E. S. Sowerby, H. J. Orr-Ewing, L. Page, E. W. Wade, G. M. Campbell.

ROYAL COLLEGE OF PHYSICIANS.—F.R.C.P.—Dr. J. A. Nixon. M.R.C.P.—Capt. F. Percival Mackie, I.M.S., F.R.C.S.

**Conjoint Board.**—*Biology:* H. R. W. Husbands, D. Munro Smith. *Medicine:* L. C. W. Baker, R. F. Bolt.

M.R.C.S., L.R.C.P. LOND.—C. H. Hart, I. R. Hudlestone, T. S. Rippon.

L.D.S. ENG.—*Physics:* K. Batten, W. A. Culverwell. *Mechanical Dentistry:* G. H. Piercy. *Dental Metallurgy:* G. H. Piercy, S. V. Shrimpton, K. Batten. *Final—Part I:* E. J. Handford, P. L. Ealand, C. H. Wilson, B. L. Morgan; *Part II:* S. Saxton, A. H. Spencer Palmer, B. W. Tyson, L. G. Kinnersley.

ATHLETIC GROUND.—The new ground at Coombe Dingle was formally opened on May 11th, when the second annual sports took place. Favoured by splendid weather, a large gathering of students and others interested in the University witnessed some excellent sports, and the students of the medical faculty again secured the inter-faculty cup with a long lead of points over the other faculties. A pavilion is in course of erection, and the cost of this has been largely helped by the donor of the field, Mr. George A. Wills.

Guild of Undergraduates.—This scheme, which has been under consideration for some time, seems to be approaching completion. At present students need not necessarily join the present Clubs Union, but under the new scheme membership will be made compulsory, and aided by an annual grant from the Council of the University, the financial basis will be a more firm one that it is at present.

Association of Alumni.—On April 25th the President and Committee of this Association entertained the members and their friends at the University, when an opportunity was given of viewing the Chemical and Physiological buildings and the equipment and general resources of these departments. They were received by the Vice-Chancellor, and a short lecture was delivered by Professor Francis. The proceedings were much enjoyed by a large gathering of alumni.

**Research Defence Society.**—A branch of this Society has been formed in Bristol, and the local secretary is Miss K. I. Williams, Llandaff House, Pembroke Vale, Clifton. A large addition to the roll of members is desired.

APPOINTMENTS.

**University of Bristol.**—H. W. Bywaters, D.Sc., has been appointed Demonstrator of Physiology.

**Bristol Royal Infirmary.**—*Senior Resident Officer:* H. Chitty, M.B., B.S. *House Surgeon:* E. W. Archer, L.M.S.S.A. *House Physicians:* S. H. Kingston, M.R.C.S., L.R.C.P., and J. S. Hopwood, M.R.C.S., L.R.C.P. *Resident Obstetric Officer:* R. S. S. Statham, M.B., Ch.B. Bristol, M.R.C.S., L.R.C.P. *House Surgeon to Ear, Throat and Nose Department:* R. C. Clarke, M.B., Ch.B. Bristol, M.R.C.S., L.R.C.P.

**Bristol General Hospital.**—*House Surgeon:* S. E. Malherbe, M.B., B.Ch. Edin. *House Physicians:* S. Marle, M.R.C.S., L.R.C.P., and H. A. Cookson, M.B., B.Ch. Edin. *Casualty House Surgeon:* F. H. Pickin, M.R.C.S., L.R.C.P.

**Children's Hospital, Bristol.**—Arthur Fells, M.B., C.M. Edin., has been appointed Assistant-Surgeon.

